# Investigation of the Structural Properties and Antioxidant Potency of Pectic Polysaccharides Derived from *Rohdea japonica* (Thunb.) Roth

**DOI:** 10.3390/molecules29174135

**Published:** 2024-08-31

**Authors:** Su Yan, Zhiying Lin, Kuo Cui, Hao Zang, Yifa Zhou, Lihui Zhang, Duo Liu

**Affiliations:** 1 School of Life Sciences, Changchun Normal University, Changchun 130032, China; yansu@ccsfu.edu.cn; 2 School of Life Sciences, Northeast Normal University, Changchun 130024, China; linzy722@nenu.edu.cn (Z.L.); cuikuo_826@126.com (K.C.); zhouyf383@nenu.edu.cn (Y.Z.); 3 School of Pharmacy and Medicine, Tonghua Normal University, Tonghua 134002, China; zanghao2013@thnu.edu.cn

**Keywords:** *Rohdea japonica* (Thunb.) Roth., rhizome, pectin, structural domain, structural elucidation, antioxidant capacity

## Abstract

This study investigated the structural composition and antioxidant properties of pectic polysaccharides extracted from *Rohdea japonica* (Thunb.) Roth. Pectins, which belong to a complex category of acidic polysaccharides, possess a wide range of biological effects stemming from their distinctive structural domains. The polysaccharides were extracted using water, and were subsequently purified through ion exchange and gel permeation chromatography. In order to elucidate their structural features, Fourier Transform Infrared Spectroscopy and Nuclear Magnetic Resonance techniques were applied. Two specific polysaccharides, WRJP-A2a and WRJP-A3b, with molecular weights of 42.7 kDa and 64.1 kDa, respectively, were identified to contain varying proportions of homogalacturonan, rhamnogalacturonan I, and rhamnogalacturonan II domains. Regarding antioxidant capacity, WRJP-A3b exhibited superior scavenging capabilities against DPPH, ABTS, and hydroxyl radicals, potentially attributed to its higher galacturonic acid content and abundance of homogalacturonan domains. These results enhance our comprehension of the structure–activity interplay of pectic polysaccharides sourced from *Rohdea japonica* (Thunb.) Roth and their potential utility in the healthcare and functional food sectors.

## 1. Introduction

Pectins, a group of complex acidic polysaccharides found in plant cells, exhibit diverse biological functions [1]. They primarily comprise the following three distinct domains: homogalacturonan (HG), rhamnogalacturonan I (RG-I), and rhamnogalacturonan II (RG-II). The HG domain, accounting for approximately 65% of total pectin content, consists of a linear polysaccharide made up of *α*-(1→4)-linked galacturonic acid (GalA) residues. Some of these GalA residues undergo methylation at the *O*-3 position and acetylation at either the *O*-2 or *O*-3 position [2]. RG-I represents 20% to 35% of total pectin, and is composed of a backbone with repeating units of [→2)-*α*-Rhap-(1→4)-*α*-GalAp-(1→], with side chains attached to the C4 position of (1→2)-*α*-Rhap. The side chains comprise arabinan, galactan, arabinogalactan-I, and arabinogalactan-II (AG-II). The percentage of neutral sugar side chains in RG-I ranges from 25% to 80%, contingent on the pectin’s source and extraction technique. Furthermore, the RG-II domain is a significantly conserved sequence found in plant cells, exhibiting remarkable uniformity among different plant species [3].

Pectins exhibit a remarkable ability to scavenge free radicals due to their high GalA content, positioning them as promising antioxidants. The unique physicochemical properties and low toxicity of pectins have attracted significant attention [4]. The macromolecular architecture of pectin imparts it with a distinct capacity to stabilize free radicals, effectively mitigating reactive oxygen species through mechanisms such as chelating metal ions and scavenging peroxides. While phenolic compounds, owing to their lower molecular weight, may exhibit swifter reaction kinetics and elevated in vitro antioxidant activity, pectin, with its high molecular weight and multifunctional structure, offers more enduring and extensive antioxidant protection within the in vivo milieu. Furthermore, pectin’s ubiquitous presence in plant cell walls, coupled with its long history of safe consumption and excellent biocompatibility, establishes a robust foundation for its safety profile [5]. As complex biological macromolecules, the antioxidant effectiveness of pectins is significantly affected by factors such as solubility, molecular weight, and structural features like monosaccharide composition, glycosidic bond type, and the sequence and configuration of sugar residues [6]. Moreover, plant pectins demonstrate universal structural characteristics alongside plant-source-specific traits [7]. Furthermore, the biological activity of pectin is greatly influenced by the composition and structural variations within its domains. Although the main chemical structure properties of pectins are well-established, subtle structural variances like side chain composition, esterification degree, molecular weight, and branching degree differ among plant species [8]. Therefore, exploring the intricate structures of different pectins is crucial for understanding the structure–activity relationship and enhancing their application in the pharmaceutical and food industries.

In recent years, there has been a surge in research focusing on pectins derived from monocotyledonous plants, which has emerged as a prominent area of scientific inquiry [9,10,11]. Among these, *Rohdea chinensis*, a plant belonging to the *Rohdea* genus, has garnered particular attention. From this plant, three distinct polysaccharides have been successfully isolated and characterized [12]. *Rohdea japonica* (Thunb.) Roth (*R. japonica*), another perennial herbaceous plant from the *Rohdea* genus, is predominantly found in various regions, including Shandong, Jiangsu, Zhejiang, Jiangxi, Hubei, Hunan, Guangxi, Guizhou, Sichuan, Anhui, Fujian, Henan, and Taiwan, along with Japan. This plant typically blooms from May to June, and bears fruit from September to November. Thriving in moist habitats, such as forest understories or grasslands at elevations between 750 and 1700 m, both its rhizome and the entire plant exhibit medicinal properties. These properties include cardiac reinforcement, diuretic effects, heat and toxin clearance, pain relief through stasis elimination, and bleeding cessation [13,14]. Despite rhodexin A being the main component of *R. japonica* [15], research on its polysaccharides remains lacking. Certainly, there exists a notable lack of research on the intricate structure–activity relationship of *R. japonica*’s pectins. Therefore, this study aims to extract pectins from *R. japonica* and ascertain their structure through a combination of chemical and instrumental analyses. Additionally, the antioxidant properties of these pectins and their related structure–activity correlations are under scrutiny.

## 2. Results and Discussion

### 2.1. Extraction of Pectic Polysaccharides from R. japonica

WRJP (the crude polysaccharide) was obtained from *R. japonica* via hot water extraction and ethanol precipitation, resulting in an 8.1% yield relative to the dry mass (Table 1). The proportions of total carbohydrates, glucuronic acid, and total protein in WRJP were 53.8%, 32.1%, and 4.2%, respectively. Monosaccharide analysis showed that WRJP consisted of GalA, Rha, Gal, Ara, Glc, GlcA, and Man in molar ratios of 29.9:4.7:39.9:7.4:15.2:1.1:1.7 (Table 1).

**Figure 1:**
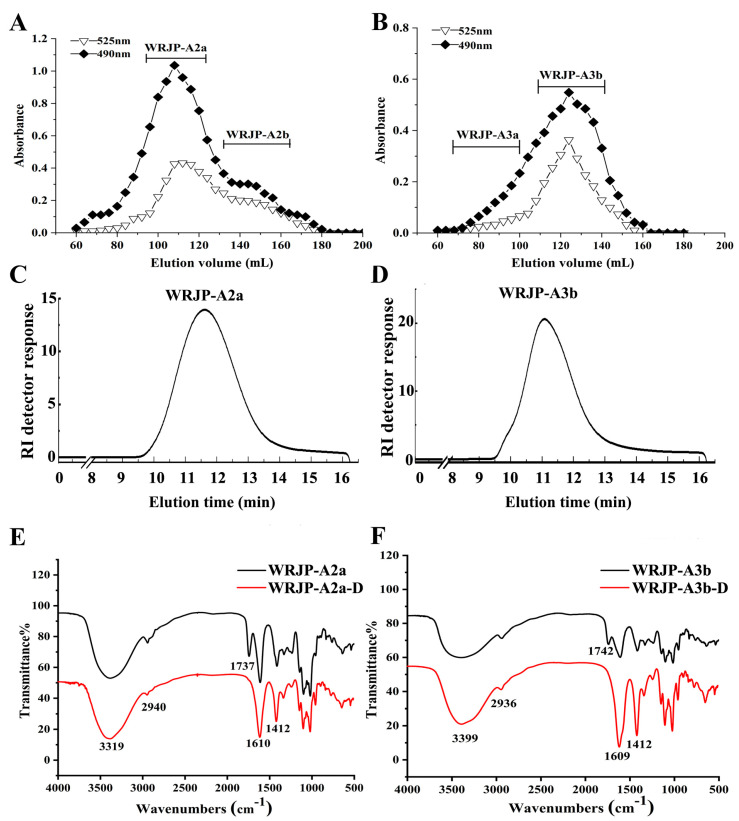
Characteristics of the WRJP-A2a and WRJP-A3b fractions. (**A**,**B**) Sepharose CL-6B elution curve, (**C**,**D**) HPGPC profiles, (**E**,**F**) FT-IR spectra.

WRJP was then separated using ion exchange chromatography to obtain a neutral fraction (WRJP-N) with a yield of 49.7% (relative to WRJP), and an acidic fraction (WRJP-A) with a yield of 26.7% (relative to WRJP). WRJP-A was further divided using a DEAE-cellulose column, resulting in WRJP-AH (7.9%), WRJP-A2 (68.2%), WRJP-A3 (10.3%), and WRJP-A5 (3.3%). Subsequently, WRJP-A2 and WRJP-A3 underwent additional purification using a Sepharose CL-6B column (Figure 1A,B), producing the following two major fractions: WRJP-A2a (59.3%) and WRJP-A3b (71.5%). The monosaccharide compositions of WRJP-A2a and WRJP-A3b were similar, primarily comprising GalA, Rha, Gal, and Ara (totaling around 95% of the total monosaccharides), with small amounts of Glc, GlcA, Xyl, and Man (Table 1). The presence of GalA, Rha, Gal, and Ara suggested the existence of HG and RG-I domains in both WRJP-A2a and WRJP-A3b. WRJP-A2a and WRJP-A3b tested positively in the TBA assay, indicating the presence of an RG-II-type pectin domain.

### 2.2. Purity, Homogeneity, and Molecular Weight of WRJP-A2a and WRJP-A3b

**Figure 2:**
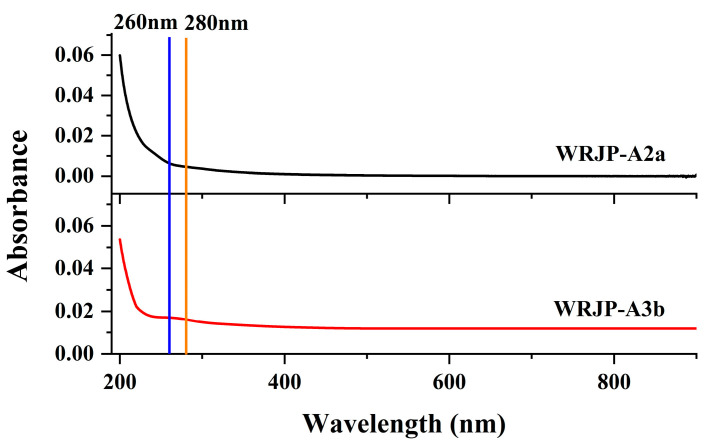
UV-vis spectra of WRJP-A2a and WRJP-A3b.

WRJP-A2a exhibited no absorbance at 260 and 280 nm, indicating the absence of nucleic acids and proteins in the fractions (Figure 2). As made evident by Figure 2, WRJP-A3b contains trace amounts of nucleic acid impurities; however, this is unlikely to significantly impact subsequent research endeavors. Therefore, the purification process resulted in highly pure pectin. High-performance gel permeation chromatography (HPGPC) was utilized to assess the homogeneity of WRJP-A2a and WRJP-A3b. Both pectin fractions displayed narrow, single symmetrical peaks in the HPGPC elution curves (Figure 1C,D), suggesting homogeneity with molecular weights of 42.7 kDa (WRJP-A2a) and 64.1 kDa (WRJP-A3b).

### 2.3. FT-IR Analysis of WRJP-A2a and WRJP-A3b

Fourier transform infrared (FT-IR) serves as a convenient and effective method for analyzing the primary structure of polysaccharides. It can be utilized to extract structural details concerning specific functional groups and sugar residue configurations within polysaccharides [16].

Both the WRJP-A2a and WRJP-A3b fractions displayed similar FT-IR spectra (Figure 1E,F). The broad and intense absorption peaks at 3319 and 3399 cm^−1^ were linked to the O−H stretching vibration characteristic of the hydrogen bonds in sugar residues. The faint absorption peaks around 2940 and 2936 cm^−1^ originated from the asymmetric C−H stretching vibrations of the −CH_3_, –CH_2_, and –CH groups. Notably, the absorption peaks near 1245, 1740, and 1610 cm^−1^ in the spectra indicated the presence of uronic acid [17]. Specifically, the peaks near 1740 cm^−1^ and 1610 cm^−1^ represented the characteristic vibrations of C = O in carboxyl groups of methyl-esterified GalA and free carboxyl groups, respectively [18]. These peaks’ areas could be leveraged to calculate the degree of methylation (DM) of acidic polysaccharides. The esterification degree of pectin could be determined using the following formula: DE (%) = [A1740/(A1740 + A1610)] × 100%, where A1740 denotes the area of esterified carboxyl groups (1740 cm^−1^) and A1610 denotes the area of free carboxyl groups (1610 cm^−1^). Employing this approach, the DM for WRJP-A2a and WRJP-A3b was calculated as 29.3% and 23.0%, respectively.

### 2.4. NMR Analysis of WRJP-A2a and WRJP-A3b

#### 2.4.1. NMR Analysis of WRJP-A2a

**Figure 3:**
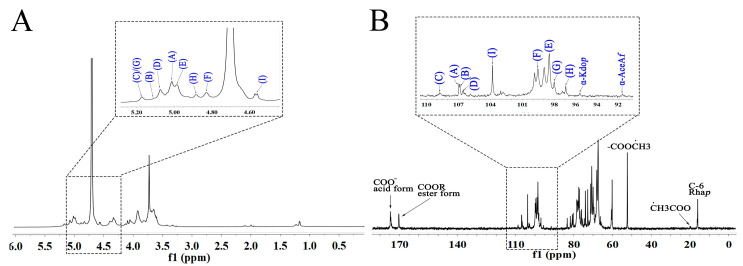
1D NMR spectra of WRJP-A2a (**A**) ^1^H spectrum and (**B**) ^13^C spectrum (A-I labeled in these spectra represent the residues corresponding to Table 2).

**Figure 4:**
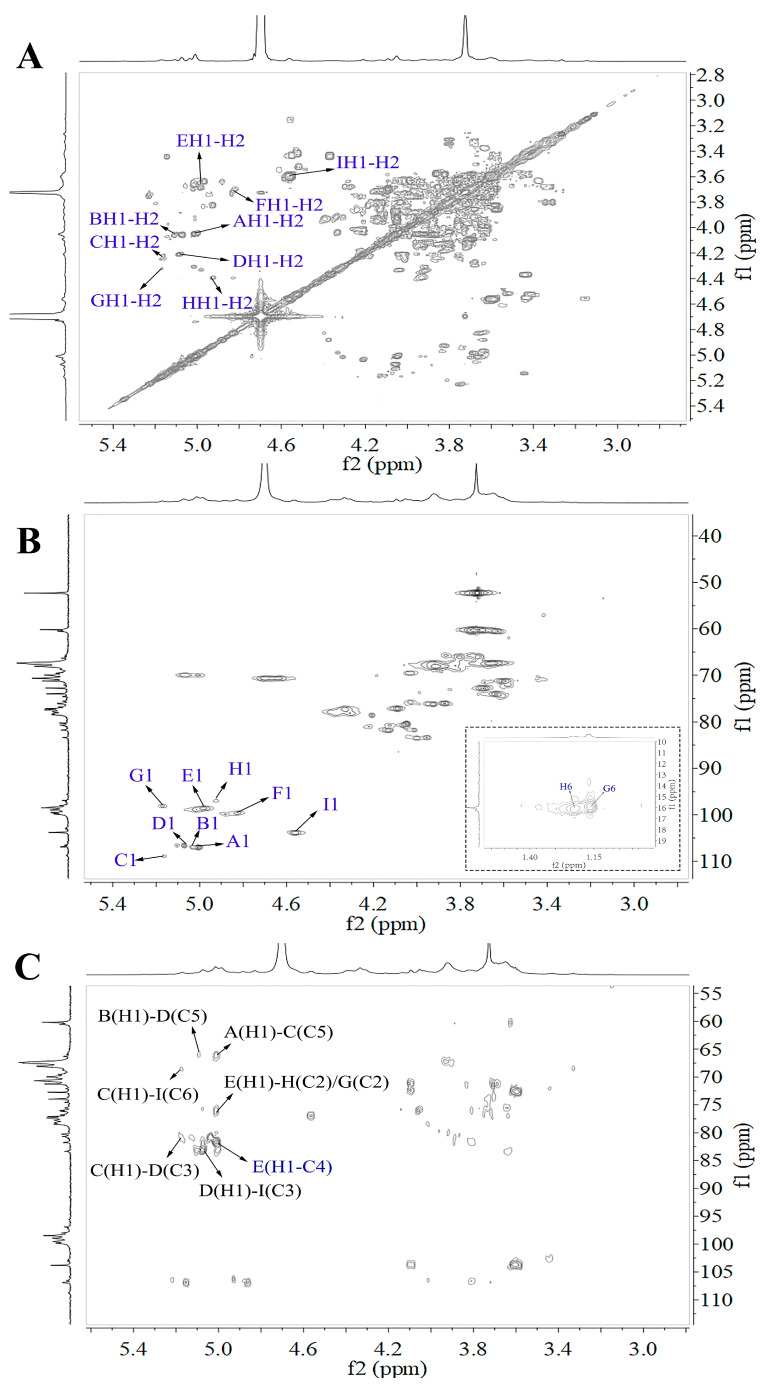
2D NMR spectra of WRJP-A2a (**A**) ^1^H-^1^H COSY spectrum, (**B**) ^1^H-^13^C HSQC, (**C**) HMBC, (A-I labeled in these spectra represent the residues corresponding to Table 2).

**Figure 5:**
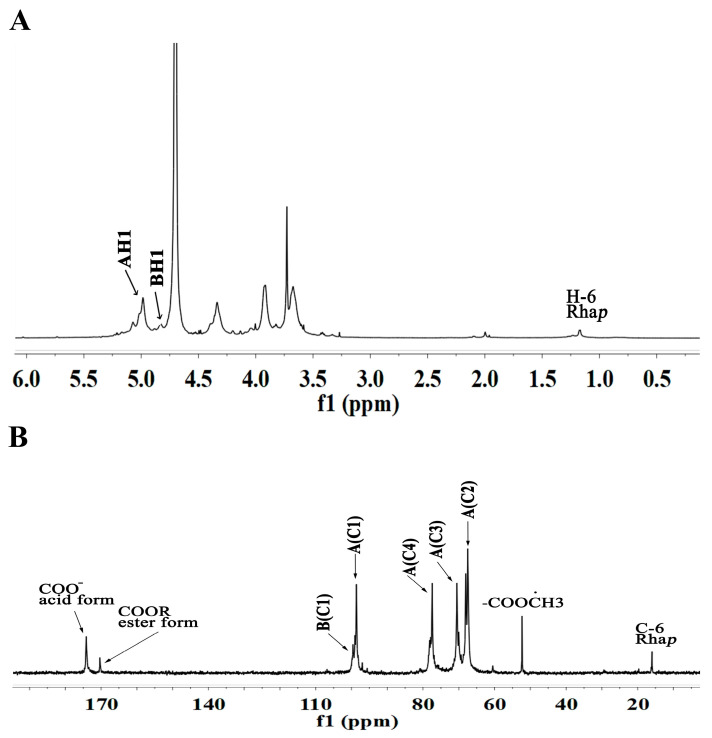
1D NMR spectra of WRJP-A3b (**A**) ^1^H spectrum and (**B**) ^13^C spectrum (A-I labeled in these spectra represent the residues corresponding to Table 3).

**Figure 6:**
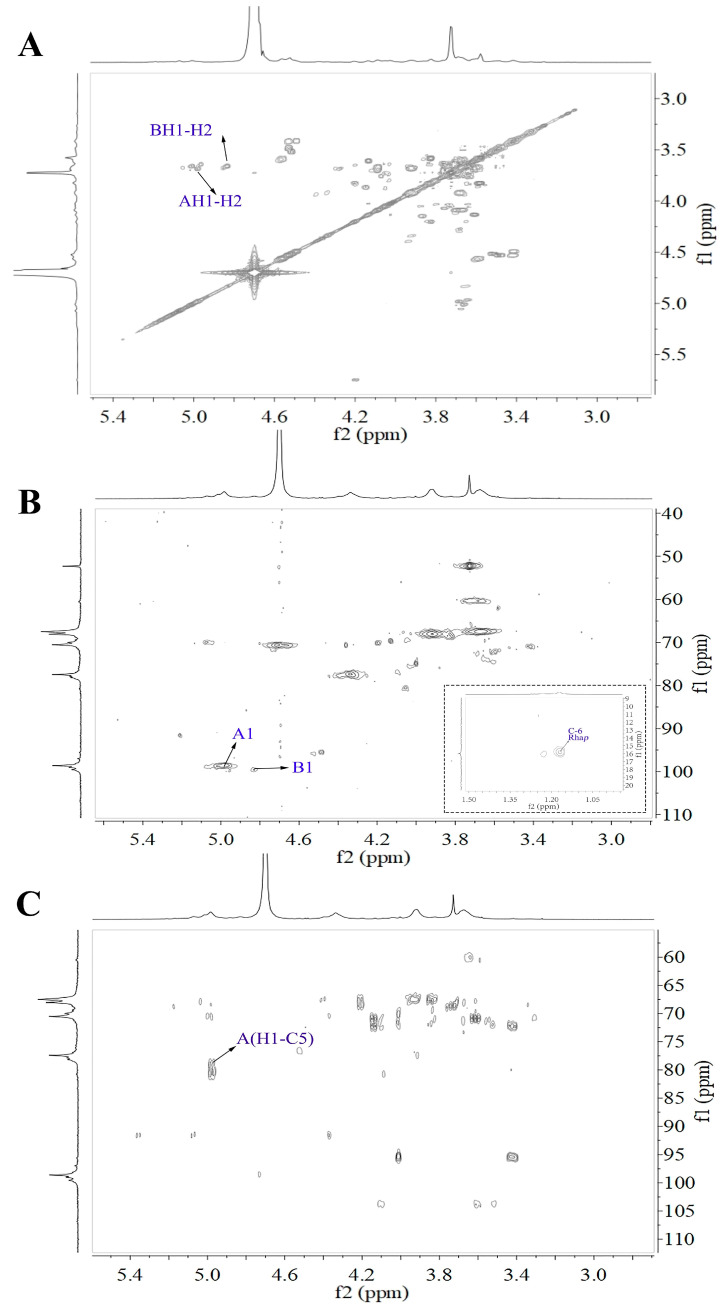
2D NMR spectra of WRJP-A3b (**A**) ^1^H-^1^H COSY spectrum, (**B**) ^1^H-^13^C HSQC, (**C**) HMBC (A and B labeled in these spectra represent the residues corresponding to Table 3).

**Figure 7:**
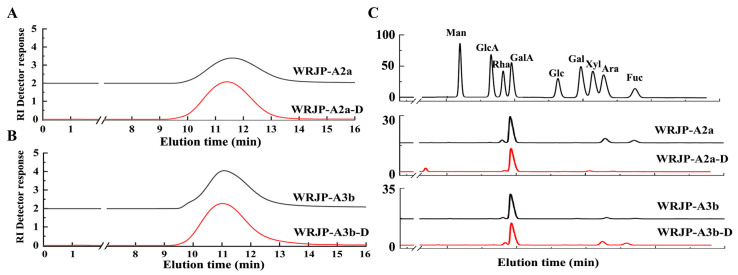
Characteristics of the de-esterified products of WRJP-A2a-D and WRJP-A3b-D. (**A**,**B**) HPGPC profiles and (**C**) monosaccharide composition.

The 1D and 2D NMR spectra of WRJP-A2a are showcased in Figure 3 and Figure 4. The anomer signals of WRJP-A2a span from 4.56 ppm to 5.16 ppm (Figure 4, Figure 5 and Figure 6A) and 91.62 ppm to 108.91 ppm (Figure 4, Figure 5, Figure 6 and Figure 7B). In the ^13^C NMR spectrum, signals at 52.47 and 19.99 ppm indicated the presence of methyl-esterified and acetylated GalA in WRJP-A2a. Additionally, signals at 95.59 and 91.62 ppm corresponded to the C2 vibration peaks of *α*-Kdop and *α*-AcefA, respectively [8], suggesting the presence of the RG-II domain in WRJP-A2a. Nine H1-C1 sugar residue-related peaks were observed in ^1^H-^13^C HSQC, with values corresponding to various residues labeled A-I. The chemical shift values for all of the carbon and hydrogen in A-I residues are detailed in Table 2. Using the same analytical method, A-I residues were identified as follows: *α*-Araf-(1→AtI, *α*-Araf-(1→AtII, (1→5)-*α*-Araf, (1→3,5)-*α*-Araf, (1→4)-*α*-GlaAp, (1→4)-*α*-GlaAp6Me, (1→2)-*α*-Rhap, (1→2,4)-*α*-Rhap, and (1→3,6)-*β*-Galp.

**Table 3:** ^1^H and^13^C NMR chemical shift assignments of the residues in WRJP-A3b.

Residues	Glycosidic Linkage		1	2	3	4	5	6
A	4)-*α*-GalA*p*-(1→	H	4.98	3.67	3.93	4.05	4.72	
		C	98.56	67.43	68.05	81.57	70.57	174.15
B	4)-*α*-GalA*p6*Me-(1→	H	4.83	3.67	3.93	4.05	4.72	
		C	99.59	67.43	68.05	81.57	70.57	170.34

We utilized the HMBC spectrum to examine the linkage pattern of A-I sugar residues in WRJP-A2a. The prominent cross peak at 4.98/81.67 ppm in Figure 4, Figure 5, Figure 6 and Figure 7C is associated with the long-range coupling signal of EH1-EC4 residues, signifying that the structure of WRJP-A2a includes the HG domain. The signal at 4.98/76.12 ppm corresponds to the cross peak of residues EH1-GC2 and EH1/HC2, indicating the connection of (1→4)-*α*-GalAp to the C2 of (1→2)-*α*-Rhap and (1→2,4)-*α*-Rhap, showing the presence of the RG-I domain in WRJP-A2a. The presence of 4.56/76.96 ppm (IH1-HC4) suggests that the branches of WRJP-A2a are linked to the C4 of (1→2,4)-*α*-Rhap in the RG-I backbone through (1→3,6)-*β*-Galp residues. Long-range coupling peaks at CH1-IC6 (5.16/68.10 ppm), DH1-IC3 (5.07/83.06 ppm), CH1-DC3 (5.16/80.42 ppm), AH1-CC5 (5.00/66.12 ppm), and BH1-DC5 (5.10/66.11 ppm) indicate the presence of AG-II side chains (A, B, C, D, and I) in WRJP-A2a, with (1→5)-*α*-*L*-Araf and (1→3,5)-*α*-Araf connected to the C3 and C6 of (1→6)-*β*-*D*-Galp in the side chain, respectively. The positioning of (1→5)-*α*-*L*-Araf is at the C5 position of (1→3,5)-*α*-Araf; hence, WRJP-A2a predominantly comprises HG and RG-I backbones with branching at the C4 of (1→2)-*α*-Rhap. A single connection passage between the WRJP-A2a side chains and its backbone links to the RG-I backbone through (1→3,6)-*β*-Galp.

#### 2.4.2. NMR Analysis of WRJP-A3b

The 1D and 2D NMR spectra of WRJP-A3b are depicted in Figure 5 and Figure 6, correspondingly. Based on the monosaccharide composition findings, WRJP-A3b demonstrates a high GalA content of 82.4%, indicating a predominance of HG-type structural domains in the pectin structure. In the ^1^H NMR spectrum (Figure 5), only signals at 4.98 and 3.83 ppm were detected in the anomer hydrogen region, ascribed to the anomer hydrogen of *α*-GalAp and *α*-GalAp6Me, respectively. The signals at 98.56 and 99.59 ppm in the ^13^C spectrum (Figure 6) are attributed to the anomer carbon of *α*-GalAp and *α*-GalAp6Me, respectively. Chemical shifts of C2-C5 are below 83 ppm (67.43 ppm, 68.05 ppm, 80.54 ppm, 70.59 ppm), indicating the presence of solely pyran-type sugar residues in WRJP-A3b. Peaks at 174.15 ppm and 170.34 ppm signify the C6 characteristic signal peaks of *α*-GalAp and *α*-GalAp6Me, respectively. Resonance peaks at 52.47 ppm and 19.69 ppm designate the characteristic signal peaks of methyl carbon in methoxy and acetyl groups, respectively. A peak at 95.78 ppm indicates the C2 vibration of *α*-Kdop, suggesting a potential minor presence of an RG-II domain in WRJP-A3b, consistent with TBA experimental outcomes.

In the ^1^H-^1^H COSY spectrum, the signals at 4.98/3.67 ppm and 3.83/3.67 ppm correspond to the H1-H2-related peaks of *α*-GalAp and *α*-GalAp6Me, respectively. The two peak signals at 4.98/98.58 ppm and 4.83/99.55 ppm in the ^1^H-^13^C HSQC spectrum represent the H1-C1 cross peaks of *α*-GlaAp and *α*-GlaAp6Me, respectively. The chemical shift values of H2/C2 to H5/C5 for *α*-GalAp and *α*-GalAp6Me are 3.67/67.43 ppm, 3.93/68.05 ppm, 4.05/80.54 ppm, and 4.72/70.57 ppm, respectively. Therefore, residues A and B correspond to (1→4)-*α*-GlaAp and (1→4)-*α*-GlaAp6Me, respectively. The signals at 1.17/15.79 ppm and 1.23/16.14 ppm represent the H6-C6 residues of Rhap. However, due to their low content, other signal peaks of this residue are not observable in the ^1^H-^13^C HSQC spectrum. Similarly, a strong signal at 4.98/80.54 ppm was observed in the HMBC spectrum (Figure 6C), indicating the presence of [→4)-*α*-GlaAp-(1→4)-*α*-GlaAp-(1→] structural units in WRJP-A3b, and suggesting the dominance of the HG domain in WRJP-A3b.

### 2.5. Enzymatic Analysis of WRJP-A2a and WRJP-A3b

Based on the monosaccharide composition and NMR results, both WRJP-A2a and WRJP-A3b contain HG and RG-I domains. WRJP-A3b is primarily HG-type pectin, while WRJP-A2a contains an AG-II domain. For a more in-depth analysis of the pectin structure and function, WRJP-A2a and WRJP-A3b were hydrolyzed using endo-PG hydrolysis, and the different domains were separated by HPGPC.

#### 2.5.1. Preparation of De-Esterified Pectin

Endo-polygalacturonase M2 (EC 3.2.1.15) specifically targets and breaks down the unesterified GalA within HG-type domains. However, the presence of methyl and acetyl groups in HG can hinder the enzymatic degradation of pectin, ultimately impacting the purity of the HG domain [19]. Consequently, this study employed a lower temperature in a mildly alkaline setting to facilitate the de-esterification of pectin. To assess the effectiveness of de-esterification and the structural integrity of WRJP-A2a and WRJP-A3b, techniques such as FT-IR, HPGPC, and monosaccharide composition analysis were utilized. Analysis of HPGPC elution profiles (Figure 7A,B) revealed no significant alterations in the molecular weight distribution of WRJP-A2a and WRJP-A3b post-saponification, suggesting that the pectin’s long chains remained unbroken during the process. Furthermore, the monosaccharide compositions of WRJP-A2a-D and WRJP-A3b-D remained largely unchanged compared to the original pectin fraction (Figure 7C). In the FT-IR spectra of WRJP-A2a-D and WRJP-A3b-D (Figure 1E,F), the disappearance of the signal near 1740 cm^−1^, attributed to the C = O bond in the methylated −COO− group, and the significant increase in the signal near 1610 cm^−1^, indicated that the methyl groups in WRJP-A2a and WRJP-A3b had been effectively removed.

#### 2.5.2. Analysis of Enzymatic Hydrolysates

**Figure 8:**
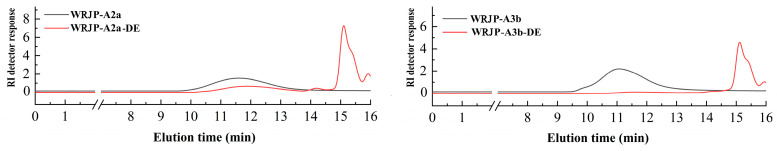
HPGPC elution profiles of the two pectins and their de-esterified enzymatic hydrolysates.

Endo-PG, a specific enzyme, has the capability to degrade unesterified GalA, leading to the breakdown of the HG-type pectin domain into oligogalacturonide structural units. It also releases RG-I- and RG-II-type pectin domains from the pectin molecules. In this study, endo-PG was employed to degrade two types of pectins, resulting in two enzymatic hydrolysates known as WRJP-A2a-DE and WRJP-A3b-DE. Analysis using HPGPC (Figure 8) revealed significant alterations in the molecular weights of WRJP-A2a and WRJP-A3b, with multiple chromatographic peaks observed in their respective profiles. To further purify these hydrolysates, a Sephadex G-75 column was utilized, leading to the preparation of three distinct types of hydrolysates (E1-E3) for both WRJP-A2a and WRJP-A3b.

The molecular weights of WRJP-A2a-DE1 and WRJP-A3b-DE1 were 50.3 kDa and 66.1 kDa, respectively, as indicated in Table 4. The primary constituents of the de-esterified hydrolysates were GalA, Rha, Gal, and Ara, with the Rha/GalA molar ratio approaching 1, suggesting that these hydrolysates belonged to the RG-I-type pectins. Notably, WRJP-A2a-DE1 contained a higher percentage of Gal (43.1%) and Ara (23.2%) compared to WRJP-A3b-DE1. The ratio of (Gal + Ara)/Rha serves as a metric for the average length and relative monosaccharide composition of the neutral side chains within the RG-I domain [19]. In WRJP-A2a-DE1, this ratio was 4.4, which was roughly 3.4 times greater than that of WRJP-A3b-DE1, suggesting that the neutral sugar side chains in WRJP-A2a-DE1 were either longer or more extensively branched. Both WRJP-A2a-DE2 and WRJP-A3b-DE2 yielded positive results in TBA reactions, confirming their classification as RG-II-type pectins. Additionally, the molecular weights of WRJP-A2a-DE3 and WRJP-A3b-DE3 were below 2.0 kDa, and their hydrolysates were predominantly composed of GalA (ranging from 97.1% to 98.4%), indicating that they were oligogalacturonides derived from the endo-PG hydrolysis of HG-type domains.

### 2.6. Antioxidant Activity Analysis

**Figure 9:**
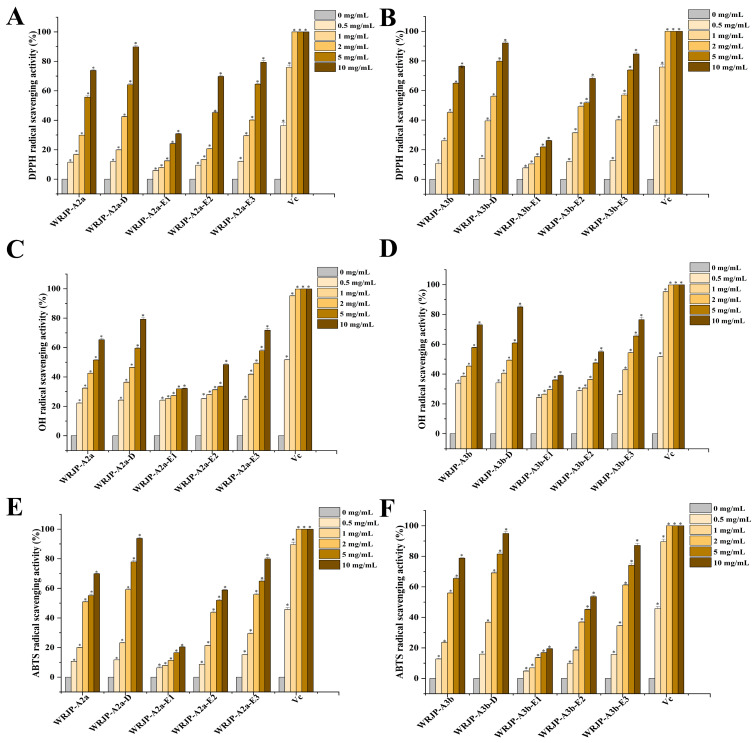
Ability of *Rohdea japonica* pectin fractions to scavenge (**A**,**B**) DPPH radicals, (**C**,**D**) hydroxyl radicals, and (**E**,**F**) ABTS radicals. *L*-Ascorbic acid was used as a positive control. Each value represents the mean ± SD (*n* = 3; * *p* < 0.05).

The antioxidant capabilities of WRJP-A2a and WRJP-A3b were assessed in vitro by evaluating their ability to scavenge DPPH, ABTS, and hydroxyl radicals. Over the tested concentration range (0.5 to 10 mg/mL), both pectins displayed a significant dose-dependent scavenging effect on these radicals, as shown in Figure 9A−F. Specifically, the Half-Maximal Inhibitory Concentration (IC_50_) values for WRJP-A2a against the three radicals were 3.07, 1.09, and 4.75 mg/mL, respectively. In comparison, the IC_50_ values for WRJP-A3b were 2.17, 1.39, and 3.27 mg/mL. These results indicate that, in the current experimental setup, WRJP-A3b exhibited a stronger radical-scavenging capacity than WRJP-A2a, although still lower than that of *L*-ascorbic acid.

To further explore the relationship between the antioxidant properties and structural composition of WRJP-A2a and WRJP-A3b, the radical-scavenging abilities of their de-esterified counterparts (WRJP-A2a-D and WRJP-A3b-D) and their various domains, including RG-I, RG-II, and oligogalacturonides, were evaluated. The level of methyl-esterification significantly influences the antioxidant activity of pectins. Specifically, the degree of methylation in pectins plays a crucial role in their antioxidant potential. Previous research has established a negative correlation between the antioxidant activity of apple pectins and their degree of methylation [20]. In our study, despite WRJP-A2a showing a higher degree of methyl-esterification compared to WRJP-A3b, WRJP-A3b displayed a more potent ability to scavenge DPPH and hydroxyl radicals. After de-esterification, both WRJP-A2a-D and WRJP-A3b-D exhibited significantly enhanced antioxidant effects compared to their non-de-esterified forms. These findings highlight the intricate relationship between pectin structure and its antioxidant activity, which may result from the interaction of multiple factors. Moreover, molecular weight is another critical factor influencing the antioxidant activity of polysaccharides. It is widely accepted that high molecular weight pectins tend to form numerous intermolecular and intramolecular hydrogen bonds, thereby reducing the accessibility and reactivity of hydroxyl groups. On the other hand, pectins with lower molecular weights may have a more relaxed conformation, allowing the exposure of free hydroxyl groups and facilitating radical-scavenging reactions [21]. Furthermore, the monosaccharide composition of polysaccharides significantly contributes to their antioxidant activity. A study has shown that GlcA and GalA have significant effects on the scavenging capabilities of *Cissus pteroclada* against DPPH, superoxide, hydroxyl, and ABTS radicals [22]. Additionally, certain neutral monosaccharides, including Gal, Ara, and Glc, have been found to have substantial impacts on the DPPH radical-scavenging ability of polysaccharides [22,23].

Another study has demonstrated that 3-*O*-methylated-*α*-*D*-galactopyranosyl present in *Pleurotus ostreatus* polysaccharides possesses antioxidant properties [24]. Pectins enriched with a certain amount of GalA are known to be potent antioxidants, and the antioxidant activity of these pectins can be attributed to the content of uronic acid and its degree of polymerization [25]. The free radical-scavenging abilities of the three domains obtained through enzymatic hydrolysis of WRJP-A2a and WRJP-A3b are summarized in Figure 9. Significant variations in radical-scavenging capabilities were observed among the domains across a dose range of 0.5–10 mg/mL. Specifically, oligogalacturonides, characterized by the highest GalA content and lowest molecular weight, exhibited the highest radical-scavenging ability, followed by the RG-II domain (E2). The RG-I domain (E1), with lower GalA content and increased branching, demonstrated the weakest radical-scavenging capability. Notably, the radical-scavenging ability of oligogalacturonides displayed a dose-dependent pattern and surpassed that of the parent pectins (WRJP-A2a and WRJP-A3b) at the same concentration. These findings align with previous research, confirming that pectins enriched with GalA and containing HG-type domains possess an enhanced radical-scavenging capacity [26].

Based on the findings, it appears that WRJP-A3b exhibits stronger antioxidant activity than WRJP-A2a, potentially attributed to its higher GalA content. Nevertheless, the presence of methyl groups and a higher molecular weight in WRJP-A3b diminished its ability to neutralize free radicals. The in vitro antioxidant properties of both WRJP-A2a and WRJP-A3b stem from the combined effects of various pectin domains, with the HG domain contributing the most significantly, followed by the RG-II domain. Conversely, the RG-I domain, characterized by a higher number of branches and molecular weight, had the least impact on the overall antioxidant activity of the pectins.

## 3. Materials and Methods

### 3.1. Materials

The rhizome material of *R. japonica* was collected from Qingdao, Shandong Province, China, in May 2022. This material originated from plants of the same age and location, ensuring uniformity. Professor Junlin Yu authenticated the specimens, and the voucher specimen is now safely stored in our laboratory’s herbarium. Prior to further processing, the rhizome material underwent minimal preparation, involving only cleaning and washing. No peeling or removal of rhizome buds was performed. Subsequently, the rhizomes were sliced and dried in a cool, well-ventilated area for future use. DEAE-cellulose and Sepharose CL-6B were sourced from GE Healthcare (United States). The monosaccharide standards were purchased from Sigma. All other chemicals utilized in the study were of analytical grade.

### 3.2. Methods

Total carbohydrate content was determined utilizing the phenol-sulfuric acid method, with a standard composed of primary monosaccharides [27]. Additionally, uronic acid content was assessed using the *m*-hydroxydiphenyl method, with GalA as the reference [28]. Ultraviolet (UV) analysis was carried out using a UV-2700 full-wavelength UV scanner (Shimadzu, Japan) to measure absorbance from 200 to 800 nm. Homogeneity and molecular weight were evaluated using a high-performance liquid chromatography (HPLC) system (Shimadzu, Japan), featuring a RID-20A UV detector and a TSKgel G3000PWXL column (7.8 cm × 30.0 cm). Detection of 3-deoxy-*D*-*manno*-2-octulosonic acid (KDO) was conducted using the thiobarbituric acid (TBA) method as detailed in the reference [29].

### 3.3. Preparation of Pectin from R. japonica

#### 3.3.1. Extraction of Pectin

*R*. *japonica* material was extracted using hot water following the outlined protocol in our library reference [8]. Initially, the dried material (1 kg) was crushed and soaked in deionized water (16 L). Extraction was conducted at 100 °C for 3 h, repeated three times under identical conditions. Subsequently, the supernatant was concentrated to 2 L at 80 °C and then treated with 8 L of 95% ethanol to precipitate the desired compounds, followed by an overnight incubation at 4 °C. The resulting precipitates underwent sequential washing with 95% ethanol and anhydrous ethanol before being dried under vacuum at 60 °C overnight. The final product, named water-soluble *R*. *japonica* polysaccharide (WRJP), represents the crude polysaccharide extracted from *R*. *japonica* material.

#### 3.3.2. Fractionation of the Pectin

WRJP (50 g) was dissolved in 1 L of deionized water until fully dissolved. Then, the sample was centrifuged and applied to a DEAE-cellulose preparative column (12 cm × 43 cm, Cl^−^ type). After a 30 min standing period, the crude polysaccharide was first eluted with 4.5 L of deionized water. The eluate was concentrated, freeze-dried, and yielded the neutral fraction called WRJP-N. Subsequently, the column was further eluted using 3 L of a 0.5 M NaCl solution, resulting in the crude pectin fraction, labeled as WRJP-A. The yields of both fractions were weighed and calculated.

**Figure 10:**
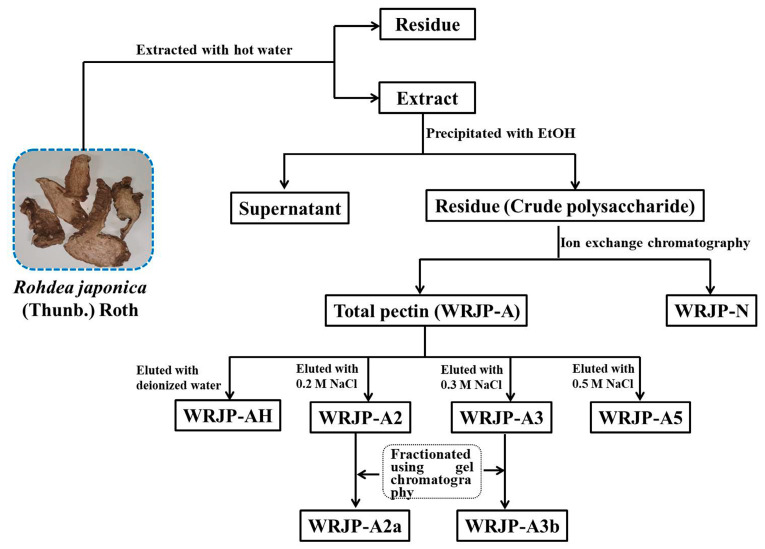
The process of isolating and purifying the pectic polysaccharides WRJP-A2a and WRJP-A3b from *Rohdea japonica*.

For WRJP-A (1 g), complete dissolution in distilled water was followed by centrifugation and loading onto a DEAE-cellulose column (12 cm × 43 cm). The raw pectin was then successively eluted with deionized water, and 0.2 M, 0.3 M, and 0.5 M NaCl solutions, maintaining a flow rate of 25 mL/min. Analysis of the total carbohydrate and uronic acid content in the eluate led to the collection and labeling of corresponding fractions as WRJP-AH, WRJP-A2, WRJP-A3, and WRJP-A5. Further purification of WRJP-A2 and WRJP-A3 was accomplished using a Sepharose CL-6B column (2.5 cm × 100 cm), where they were eluted with a 0.15 M NaCl solution at a flow rate of 0.5 mL/min. This procedure resulted in two purified pectic polysaccharides, named WRJP-A2a and WRJP-A3b. Figure 10 summarizes the extraction and fractionation method for obtaining pectins from *R. japonica*.

### 3.4. Chemical Characterization Analysis

A sample of polysaccharide ranging from 2 to 4 mg underwent hydrolysis using a 2 M anhydrous HCl-methanol solution and trifluoroacetic acid, following established protocols [30]. The hydrolyzed polysaccharide was then treated with 1-phenyl-3-methyl-5-pyrazolone (PMP) at 70 °C for 30 min. The resulting PMP derivatives were purified via chloroform extraction and analyzed using an HPLC system, comprising an SPD-20A UV-visible diode-array detector, and a COSMOSIL 5C18-PAQ column. The mobile phase consisted of a 0.1 mol/L PBS solution (pH 6.9) with 17% acetonitrile (*v*/*v*). Set parameters included a column temperature of 35 °C, a detection wavelength of 245 nm, a flow rate of 1 mL/min, and an injection volume of 10 μL.

### 3.5. FT-IR Spectroscopy

Fully dried samples (2 mg) were mixed thoroughly with potassium bromide at a 1:100 (*w*/*w*) ratio and analyzed using a Spectrum Two FT-IR spectrometer (PE, USA) within the spectral range of 4000 to 400 cm^−1^.

### 3.6. Nuclear Magnetic Resonance Analysis

For the NMR analysis, 20 mg samples were dissolved in 0.5 mL of D_2_O (99.9% purity). The ^1^H NMR, ^13^C NMR, ^1^H-^1^H COSY, ^1^H-^13^C HSQC, and HMBC spectra were recorded using a Bruker AV600 MHz NMR spectrometer (Germany) at a temperature of 25 °C.

### 3.7. De-Esterification and Enzymatic Hydrolysis

To produce the de-esterified pectins, 500 mg of WRJP-A2a and WRJP-A3b were separately dissolved in 15 mL of distilled water. The solutions were mixed thoroughly and then pre-cooled at 4 °C for 6 h. Subsequently, 15 mL of pre-cooled 0.2 M NaOH solution was slowly added to each sample, and the mixtures were incubated at 4 °C for 4 h with gentle stirring [8]. The reaction solutions were neutralized to a pH of 7.0 using 10% glacial acetic acid, desalted on a Sephadex G-10 column, and, finally, freeze-dried to obtain the desired de-esterified pectins (WRJP-A2a-D and WRJP-A3b-D).

WRJP-A2a-D and WRJP-A3b-D were dissolved in a 50 mM acetic acid-ammonium acetate solution adjusted to pH 5.0, achieving a concentration of 1 mg/mL. In the same solution, 50 μL of endo-polygalacturonase M2 (EC 3.2.1.15) was added, and the mixture was incubated at 40 °C for 24 h. Following this, the reaction solution underwent heating in a boiling water bath for 15 min to deactivate the pectinase. Enzymatic hydrolysates were separated using a Sephadex G-75 column (2.6 cm × 100 cm) and eluted with 0.15 M NaCl at a flow rate of 0.4 mL/min. The corresponding eluent fractions were collected, desalted through a Sephadex G-10 column, and freeze-dried. Three subfractions each were obtained from WRJP-A2a and WRJP-A3b, designated as WRJP-A2a-D-E1 to E3 and WRJP-A3b-D-E1 to E3, respectively.

### 3.8. Antioxidant Activity Assay

#### 3.8.1. DPPH Radical-Scavenging Activity

The pectin fractions’ scavenging ability towards the DPPH radical was assessed using previously established methods [31].

In this procedure, 500 μL of pectin solution with varying concentrations (0.5, 1, 2, 5, and 10 mg/mL) was mixed with 2 mL of 0.5 mM DPPH solution. The mixture was then incubated in darkness for 30 min, followed by absorbance measurement at 517 nm. *L*-ascorbic acid was used as a positive control for comparison, while ultrapure water and an equal volume of anhydrous methanol (in place of the DPPH solution) served as blank controls. The DPPH-scavenging activity of the fractions was determined using the provided formula, as follows:

(1) $$\%scavenging=\left(1-\frac{A_{sample}-A_{control}}{A_{blank}}\right)\times 100\%$$

*A_sample_*: This represents the absorbance value recorded for the sample solution.

*A_control_*: This represents the absorbance value recorded for the background solution, which was prepared using anhydrous methanol instead of the DPPH solution.

*A_blank_*: This represents the absorbance value recorded for the blank control.

#### 3.8.2. ABTS Radical-Scavenging Activity

The scavenging capability of the pectin fractions towards ABTS radicals was evaluated using a previously established method [32]. Freshly prepared daily, the ABTS working solution was used. To assess the ABTS radical-scavenging ability of the fractions, 400 μL of the sample solution at various concentrations (0.5, 1, 2, 5, and 10 mg/mL) was thoroughly mixed with 400 μL of the ABTS working solution in a reaction tube. The reaction was carried out in a dark environment at 25 °C for 30 min. The absorbance was then recorded at 732 nm. As a control, an equal volume of ultrapure water was used instead of the sample solutions. The ABTS radical-scavenging activity of the fractions was determined using the provided formula, as follows:

(2) $$\%scavenging=\left(1-\frac{A_{sample}-A_{control}}{A_{blank}}\right)\times 100\%$$

*A_sample_*: This represents the absorbance value recorded for the sample solution.

*A_control_*: This represents the absorbance value recorded for the background solution, which was prepared using anhydrous methanol instead of the DPPH solution.

*A_blank_*: This represents the absorbance value recorded for the blank control.

#### 3.8.3. Hydroxyl Radical-Scavenging Activity

The pectin fractions’ scavenging capacity towards hydroxyl radicals was evaluated using a previously established method [33].

For this, 100 μL of the pectin solution at various concentrations (0.5, 1, 2, 5, and 10 mg/mL) was combined with an equal volume of FeSO_4_ solution (9.0 mM), and a salicylic acid solution in absolute ethanol (9.0 mM). This mixture was then reacted with 100 μL of H_2_O_2_ solution (8.8 mM) in a reaction tube maintained at 25 °C for 30 min, with an absorbance measurement at 532 nm. Ultrapure water was used as a control instead of the pectin, and *L*-ascorbic acid was the positive control. An equal volume of ultrapure water replaced the H_2_O_2_ solution as an additional control. The hydroxyl radical-scavenging capacity was determined using the provided formula, as follows:

(3) $$\%scavenging=\left(1-\frac{A_{sample}-A_{control}}{A_{blank}}\right)\times 100\%$$

*A_sample_*: This represents the absorbance value recorded for the sample solution.

*A_control_*: This represents the absorbance value recorded for the background solution, which was prepared using anhydrous methanol instead of the DPPH solution.

*A_blank_*: This represents the absorbance value recorded for the blank control.

### 3.9. Statistical Analysis

The results pertaining to the antioxidant effect are presented as the mean ± standard deviation. A one-way analysis of variance, followed by post hoc least significant difference tests, was employed to assess significant differences among groups. Each assay was replicated three times to ensure consistency. The gathered experimental data were subjected to analysis using IBM SPSS software (version 23.0).

## 4. Conclusions

The primary aim of this research was to undertake a comprehensive examination of the structural characteristics and antioxidant potential of two unique pectins, namely WRJP-A2a and WRJP-A3b, which were isolated from *R. japonica*. WRJP-A2a and WRJP-A3b were obtained through multiple purification steps. Their respective molecular weights are 42.7 kDa and 64.1 kDa. The primary monosaccharides identified in both pectins, comprising over 95% of their total composition, are GalA, Rha, Gal, and Ara. Further analysis of their structural composition revealed the presence of the following three distinct domains: HG, RG-I, and RG-II, each with varying mass ratios. Specifically, WRJP-A2a exhibited a ratio of 16.75:6.75:1.00, whereas WRJP-A3b showed a ratio of 28.00:3.67:1.00. Notably, both pectins were predominantly composed of HG domains, with WRJP-A2a containing 67.0% and WRJP-A3b containing 84.6% HG domains, respectively. Utilizing FT-IR spectroscopy, the primary structure and the degree of methylation of the two pectins were analyzed. This was followed by an in-depth investigation using 1D and 2D NMR technologies, which revealed the complex linkage patterns between their sugar residues. A subsequent thorough analysis of the de-esterified products and their corresponding enzymatic hydrolysates of WRJP-A2a and WRJP-A3b provided valuable insights into their structural characteristics. Furthermore, notable differences in methyl-esterification levels were detected between the two pectins, resulting in varied radical-scavenging abilities. Specifically, WRJP-A3b, which boasts higher content of GalA and HG domains, displayed remarkable scavenging activity against DPPH, ABTS, and hydroxyl radicals. This exceptional antioxidant performance renders WRJP-A3b a promising candidate for use as a natural antioxidant, with potential applications in the production of functional foods and pharmaceuticals.

## Figures and Tables

**Table 1:** Yield, molecular weight, and monosaccharide composition of pectic polysaccharides extracted from *Rohdea japonica*.

	WRJP	WRJP-N	WRJP-A	WRJP-A2a	WRJP-A3b
Yield (w%)	8.1 ^a^	49.7 ^b^	26.7 ^b^	40.4 ^c^	6.6 ^c^
Molecular weight (kDa)		ND	ND	42.7	64.1
Monosaccharide Composition					
GalA	29.9	NONE	55.2	60.9	82.4
Rha	4.7	NONE	5.9	6.7	4.8
Gal	39.9	72.6	24.4	19.9	5.1
Ara	7.4	4.0	10.9	10.7	2.9
Glc	15.2	21.4	1.0	0.3	1.1
GlcA	1.1	NONE	0.4	0.8	1.5
Xyl	NONE	NONE	0.9	NONE	1.0
Man	1.7	2.0	0.3	0.6	1.2

^a^ Yield relative to the dry weight of the plant material. ^b^ Yield relative to WRJP. ^c^ Yield relative to WRJP-A. ND: Not detected.

**Table 2:** ^1^H and ^13^C NMR chemical shift assignments of the residues in WRJP-A2a.

Residues	Glycosidic Linkage		1	2	3	4	5	6
A	*α*-Ara*f*-(1→ ^AtI^	H	5.01	4.00	3.86	4.06	3.65	
		C	106.98	83.36	76.00	80.47	60.46	
B	*α*-Ara*f*-(1→ ^At Ⅱ^	H	5.10	4.03	3.93	4.06	3.74	
		C	106.64	83.24	76.18	80.47	60.34	
C	→ 5)-*α*-Ara*f*-(1 →	H	5.16	4.10	4.08	4.22	3.72	
		C	108.91	81.02	77.11	81.02	66.12	
D	→ 3,5)-*α*-Ara*f*-(1 →	H	5.07	4.21	4.03	4.00	3.80	
		C	106.60	78.55	83.24	83.46	66.11	
E	→ 4)-*α*-GalA*p*-(1 →	H	4.98	3.65	4.03	4.02	4.64	
		C	98.57	67.40	69.59	81.67	70.67	174.62
F	→ 4)-*α*-GalA*p6*Me-(1 →	H	4.83	3.72	3.91	4.01	4.70	
		C	99.61	66.12	68.10	81.75	69.67	170.32
G	→ 2)-*α*-Rha*p*-(1 →	H	5.17	4.33	4.03	3.33	3.65	1.16
		C	98.06	76.12	69.59	71.37	67.40	15.97
H	→ 2,4)-*α*-Rha*p*-(1 →	H	4.92	4.39	3.99	3.93	3.65	1.23
		C	96.98	76.12	73.64	76.96	67.40	16.12
I	→ 3,6)-*β*-Gal*p*-(1 →	H	4.56	3.60	4.00	4.03	3.63	3.91
		C	103.87	72.40	83.06	69.59	74.00	68.10

**Table 4:** Yield, molecular weight, and monosaccharide composition of enzymatic hydrolysates (E1–E3 fractions) of the pectins WRJP-A2a and WRJP-A3b.

Fractions	Yield ^a^ (%)	TBA Test	Molecular Weight (kDa)	Monosaccahride Composition (mol%)							
				GalA	Rha	Gal	Ara	Glc	GlcA	Man	Xyl
WRJP-A2a-DE1	27.6	-	50.3	15.7	15.1	43.1	23.2	1.1	0.8	0.8	0.2
WRJP-A2a-DE2	4.4	+	4.9	35.0	14.3	17.5	21.4	3.5	5.4	1.2	1.7
WRJP-A2a-DE3	67.0	-	<2.0	98.4	0.3	0.3	-	0.6	-	-	0.4
WRJP-A3b-DE1	11.2	-	66.1	27.4	27.2	21.4	15.3	5.1	1.8	1.8	-
WRJP-A3b-DE2	3.2	+	4.8	38.3	15.4	17.5	13.2	5.9	4.4	2.4	2.9
WRJP-A3b-DE3	84.6	-	<2.0	97.1	-	-	0.5	0.6	-	1.4	0.4

^a^ Yield relative to WRJP-A2a or WRJP-A3b.

## Data Availability

Data are contained within the article.
